# Temporal Association Between Vertebral Artery Dissection and SARS-CoV-2 Infection and Vaccination: A Case Report

**DOI:** 10.7759/cureus.107521

**Published:** 2026-04-22

**Authors:** Victor Moas, Mack Qin

**Affiliations:** 1 Surgery, Duke University Medical Center, Durham, USA; 2 Surgery, Indiana University Health, Indianapolis, USA

**Keywords:** arterial dissection, covid-19, covid vaccine, ischemic, ischemic stroke, mrna vaccine, stroke, vertebral artery dissection

## Abstract

Since the emergence of severe acute respiratory syndrome coronavirus 2 (SARS-CoV-2), a broad range of neurologic and vascular complications have been described, including ischemic stroke and arterial thrombosis. We report the case of a 39-year-old previously healthy male active-duty US Navy submariner who developed a right vertebral artery dissection complicated by posterior circulation ischemic stroke following symptomatic COVID-19 infection and subsequent mRNA vaccination. Nine months after SARS-CoV-2 infection confirmed on antigen testing, and three weeks after completing the second dose of the Moderna mRNA-1273 vaccine, the patient presented with transient focal neurologic deficits and headache. Computed tomography angiography demonstrated dissection and proximal occlusion of the right vertebral artery with distal reconstitution via thyrocervical trunk collaterals. Magnetic resonance imaging revealed an acute right inferior cerebellar infarct in the posterior inferior cerebellar artery distribution. The patient was managed conservatively with dual antiplatelet therapy and statin therapy, with gradual symptom resolution following physical therapy. This case highlights a potential association between SARS-CoV-2 infection, vaccination, and delayed vascular pathology, possibly mediated by endothelial dysfunction and prothrombotic states. Clinicians should consider maintaining a heightened index of suspicion for vascular etiologies in patients presenting with neurologic symptoms following COVID-19 infection or vaccination, particularly in younger individuals without traditional risk factors.

## Introduction

In March 2020, the World Health Organization (WHO) declared the SARS-CoV-2 outbreak to be a pandemic [1]. The virus has been known to affect multiple organ systems, with neurologic sequelae like headaches reported in 11-34% of hospitalized COVID-19 patients, and an increase in micro-clots and cardiovascular complications in the post-illness phase [2-4]. Additionally, arterial dissections following COVID-19 have been identified in the coronary, cerebral, vertebral, cervical, renal, and splanchnic arteries [5]. We present the first case of a vertebral artery dissection (VAD) and acute stroke following symptomatic SARS COV-2 infection and subsequent vaccination in a previously healthy adult male submariner.

## Case presentation

A 39-year-old male US Navy Submariner of South Asian ancestry with longstanding (more than five years) hypertension for which he took losartan and hydrochlorothiazide contracted a mild case of COVID-19 (diagnosed on antigen testing) following a transit from the East Pacific to Kittery, ME, in October of 2020. Symptoms included moderate to severe cough, sore throat, chills, and myalgias, and were managed in the outpatient setting with antipyretics and phenylephrine. The patient’s acute symptomatic phase lasted at least six days, with self-limited episodes of intermittent dizziness that persisted for several months after. In early 2021, the patient received the first dose of the Moderna Spikevax vaccine series, and in March, he received the second dose without any immediate post-vaccination complications. On May 6, 2021, nine months after infection, and three weeks after receiving the second dose of the vaccine, the patient developed a sudden onset of dizziness and left lower extremity weakness, which resolved within minutes. This coincided with a mild right-sided headache. On presentation to an outpatient clinic, the neurologic exam was initially unremarkable, but the patient reported another sudden bout of dizziness and left upper (mild motor drift) and lower extremity weakness (National Institutes of Health Stroke Scale (NIHSS) score of 2), which raised concern for a transient ischemic attack. This second bout of symptoms also resolved within minutes. On exam, cranial nerves 2-12 were intact; gait was normal; Romberg was negative; sensorium was grossly intact to light touch on the back, trunk, and extremities. Strength was normal and symmetric in all extremities, no hyperreflexia was noted, and coordination was noted to be intact on finger/nose, rapid alternating movements, and heel/shin. He was referred to the emergency department (ED) for further evaluation.

**Figure 1:**
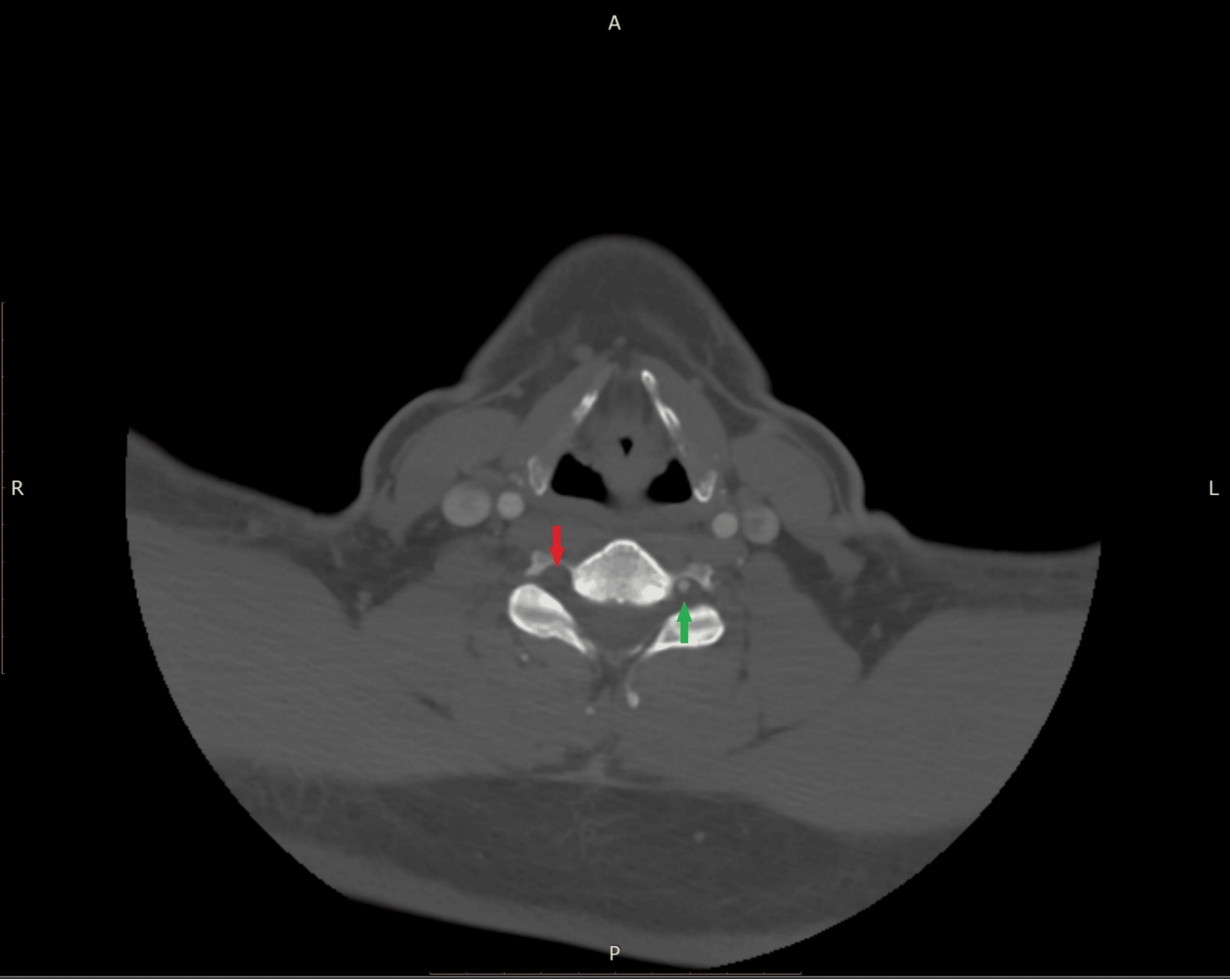
Axial view of a CT angiogram obtained on the day of presentation demonstrating no passage of contrast through the right transverse foramen (red arrow). The passage of contrast is observed through the left transverse foramen (green arrow).

**Figure 2:**
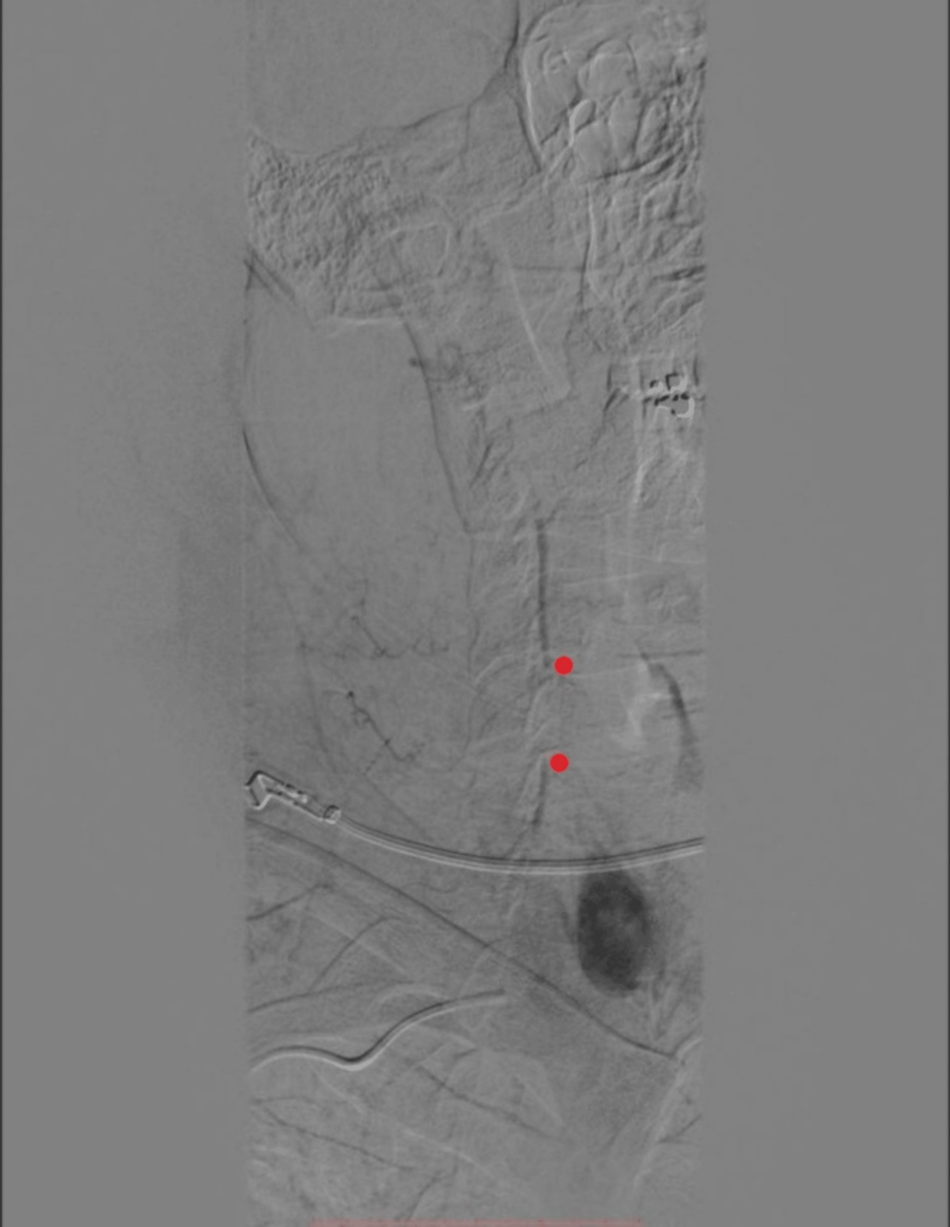
Fluoroscopy obtained on the day of presentation demonstrating lack of contrast passage across the proximal right vertebral artery, with backflow across the distal vertebral artery; the gap in flow is denoted by red dots.

**Figure 3:**
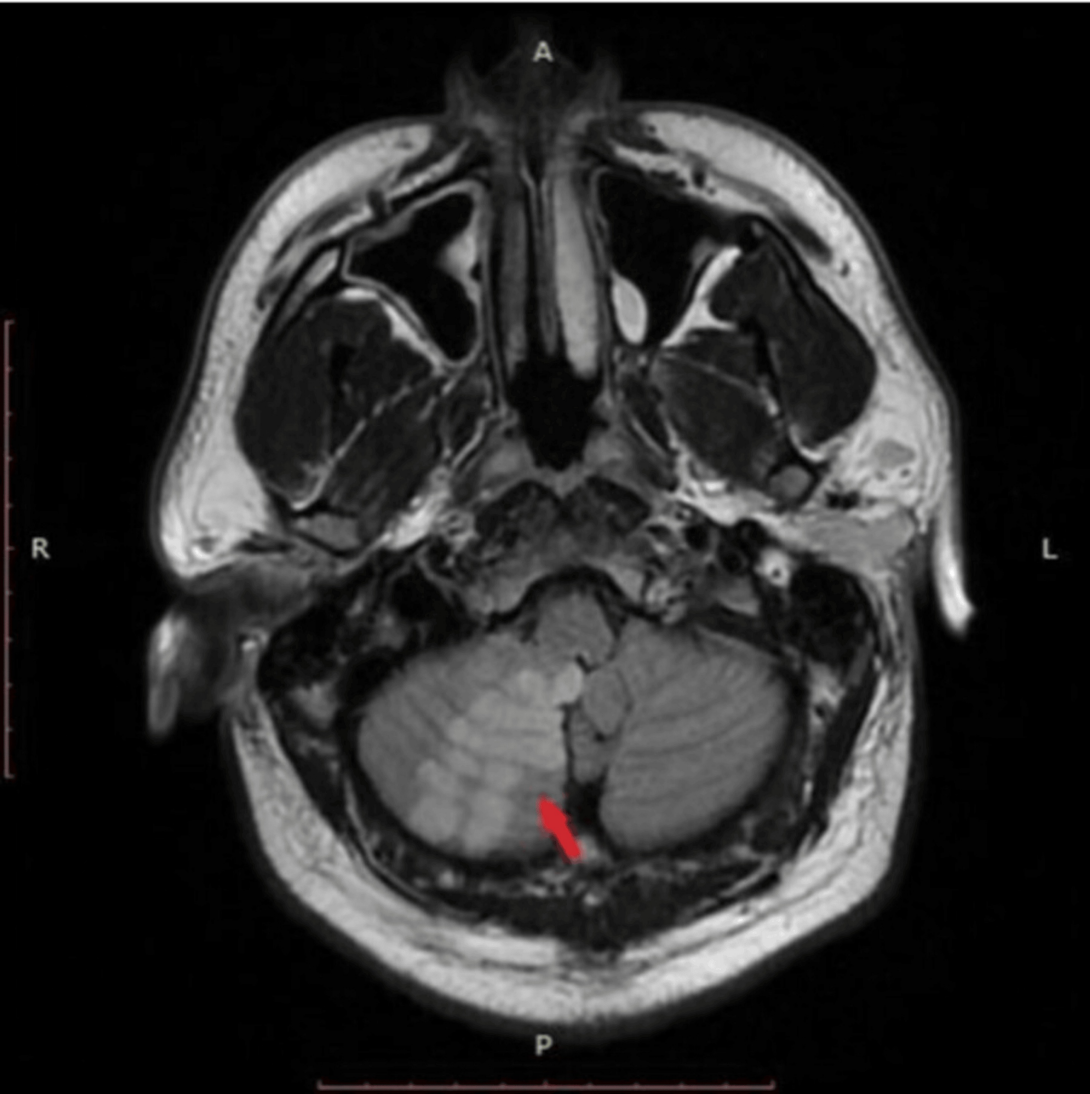
Area of ischemia noted roughly 36 hours after initial presentation in the cerebellum (red arrow) on T2-FLAIR MRI imaging (axial view). The affected area demonstrating higher intensity signal owing to increased amount of fluid from reactive edema. FLAIR: fluid-attenuated inversion recovery

At the ED, computed tomography with angiography of the head and neck identified dissection of the right vertebral artery (Figure 1), noting minimal residual flow from small vessel branches of the right thyrocervical trunk feeding the distal right vertebral vessel. Catheter angiography revealed a lack of contrast passage across the proximal right vertebral artery with backflow across the distal vertebral artery (Figure 2). The patient underwent MRI of the head with and without contrast within the same hospitalization, which noted an acute infarct of the inferior right cerebellar hemisphere in the distribution of the posterior inferior cerebellar artery (PICA) (Figure 3). The patient was admitted for further workup and observation.

Follow-up history was negative for cervical manipulation, recent excessive strain, or head/neck trauma; and the patient denied use of any steroids, illicit drugs, alcohol, or tobacco. No autoimmune, thrombophilic, or connective tissue disorder screening was performed. Follow-up neurologic exams while inpatient were unremarkable. He was discharged without neurologic deficits (modified Rankin score of 0) two days after admission on aspirin 81 mg daily, clopidogrel 75 mg daily, and a high-dose statin.

**Figure 4:**
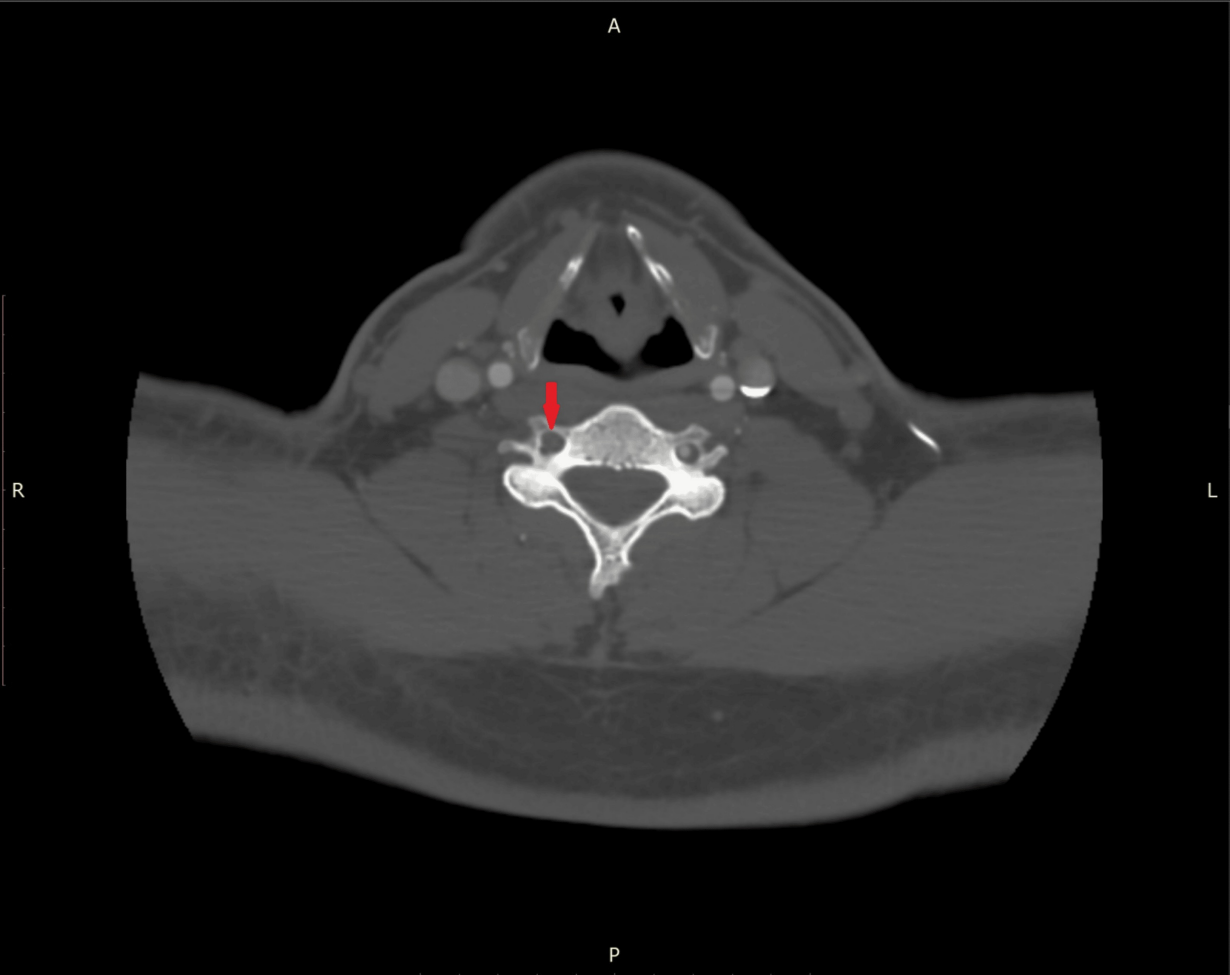
Repeat CT angiography obtained six weeks from presentation demonstrating persistent non-opacification (red arrow) of the right vertebral artery.

At follow-up with neurointerventional radiology, no intervention on the occluded vessel was recommended, and dual antiplatelet therapy was continued. He subsequently followed up with neurology one month after initial presentation, without recurrence of his prior symptoms. Repeat CT angiogram of the head and neck was obtained six weeks from presentation and was suggestive of subacute occlusion, revealing proximal occlusion of the right vertebral artery with reconstitution at the level of the mid cervical spine via thyrocervical trunk branches and muscular branches, as well as an evolving infarct in the right inferior cerebellum in the vicinity of the prior right PICA infarct (Figure 4). This evolving infarct was later noted to have become symptomatic, with the patient having developed persistent disequilibrium, reportedly waxing and waning, on follow-up with his primary care manager 2.5 months after presentation. These symptoms were mild, having no effect on his ability to carry out activities of daily living or participate in shoreside work (modified Rankin score of +1). On follow-up nine months from initial presentation, the patient was found to be in a normal state of health, his symptoms having resolved after completing a course of physical therapy targeted at improving balance (modified Rankin score of 0). He completed a six-month course of dual antiplatelet therapy, following which he remained on low-dose aspirin only. His recovery and lack of residual symptoms eventually allowed him to remain on active duty in the US Navy.

## Discussion

Vascular dissection occurs when tears in the tunica intima allow blood to infiltrate and delaminate the tunica intima from the underlying media or outer adventitia. Spontaneous VAD can lead to ischemic stroke secondary to subsequent thromboembolism or from luminal narrowing caused by the intimal flap [3]. Symptoms can range from severe neck pain to focal neurologic deficits, including dizziness, vertigo, diplopia, ataxia, and dysarthria [4].

Contributing factors for vascular dissections range from genetic predisposition, as in the case of connective tissue disorders like Ehlers-Danlos syndrome and Marfan syndrome, to external factors like trauma or chiropractic manipulation of the neck. In this patient, no genetic screening was performed. Other potential risk factors include tobacco use, hypertension, and oral contraceptive use [3].

Even prior to the emergence of COVID-19, infectious etiologies have been postulated as a potential risk factor for spontaneous VAD, with vigorous neck hyperextension or rotation during coughing or sneezing proposed as the mechanism [3]. For the hepatitis viruses (excepting hepatitis D), a causal relationship is thought to exist between infection and vasculitis, with proposed mechanisms ranging from direct vascular invasion to immune complex-mediated vessel damage [6,7]. In COVID-19, dissections have been noted throughout the arterial system, from the aorta to smaller vessels such as the cervical arteries [8-10]. Post-mortem investigations in COVID-19 positive patients have identified immune-mediated endothelial dysfunction and even noted the presence of viral inclusion bodies in endothelial cells [8,11]. This phenomenon of endotheliitis and subsequent vasculitis predisposing to thrombosis suggests a pathway that could explain direct vascular damage by COVID-19 [8,11]. Interestingly, SARS Co-V2’s known binding to the angiotensin-converting enzyme 2 (ACE2) receptor, ubiquitous on the endothelial lining, provides a direct link between COVID-19 and resultant endothelial damage [12,13].

In the absence of population-level data, an increased risk of vascular complications cannot be determined, but analyses suggest the all-cause incidence of stroke in patients hospitalized with COVID-19 to be 1-2%, with a relative risk of 1.3 relative to patients hospitalized without a diagnosis of COVID-19 [14]. Cross-sectional analysis of vascular function in young adults after COVID-19 demonstrates poorer vascular function and higher arterial stiffness at three to four weeks following positive testing [15]. Another study noted that increased D-dimer levels were observed in 25.3% of patients up to four months after infection, but other coagulation markers, such as prothrombin time and activated partial thromboplastin time, returned to normal in >90% of patients [16]. Taken together, these studies suggest increased risk in at least two of Virchow’s triad (intravascular disruption and hypercoagulable state), which could help explain any increased risk of thromboembolism post-infection.

Vascular effects of the COVID-19 mRNA vaccines have also been documented. Terentes-Printzios et al. described that impaired endothelial function, as measured by diminished flow-mediated vascular dilation (FMD) and an increase in serum inflammatory markers 24 hours following the second dose of the BNT162b2 vaccine, returned to baseline by 48 hours, and Yamaji et al. noted that the same decrease in FMD persisted two weeks after the second dose of the BNT162b2 vaccine [17,18]. On repeat testing six months following vaccination, FMD was found to have normalized [18]. The extent to which these findings indicate any clinically significant increased risk of vascular complications remains yet to be determined, and may warrant further study.

Special consideration was given to the patient’s status as an active duty submariner. Given the resource limitations and evacuation difficulties inherent to submarine duty, the decision was made to remove the patient from future deployments while allowing him to participate in shoreside duties relevant to his rating.

The patient’s unique atmospheric exposure while shipboard could be raised as an additional consideration in this case. During normal operations, the submarine atmosphere can consist of a reduced concentration of oxygen, ranging from anywhere from 21% to 18%, depending on operational constraints. Other gases, such as carbon dioxide, can also vary relative to standard atmospheric gas composition [19]. In the case of this particular patient, however, his last exposure to the submarine environment was several months prior to initial infection, and he had also been breathing standard atmospheric gas in the period between initial infection and development of his vertebral dissection.

## Conclusions

We describe a patient with VAD leading to a PICA stroke following COVID-19 and subsequent vaccination. While mechanisms have been proposed for COVID-19 and vaccine-mediated endothelial damage and dysfunction, long-term vascular effects have yet to be fully defined. It should be stated that we present only a temporal association for the purpose of hypothesis-generation. Though no causal conclusions should be drawn from this case, we recommend clinical vigilance in patients presenting with neurologic syndromes following COVID-19 or mRNA vaccination.
